# Post‐Botulinum Headache in Cosmetic Practice: A Prospective Study

**DOI:** 10.1111/jocd.70731

**Published:** 2026-02-18

**Authors:** Ümit Akpınar, Omer Vural, Ekrem Civas, Alper Koycu

**Affiliations:** ^1^ Private Dermatology Practice Ankara Türkiye; ^2^ Department of Otolaryngology Head and Neck Surgery Faculty of Medicine, Kafkas University Kars Türkiye; ^3^ Department of Otolaryngology Head and Neck Surgery, Faculty of Medicine Baskent University Ankara Türkiye

## Abstract

**Background:**

Botulinum toxin is widely used for aesthetic and functional indications and is generally considered safe; however, post‐procedural headache remains a recognized but underexplored adverse effect, particularly in cosmetic practice.

**Objective:**

To evaluate the incidence, characteristics, and potential risk factors of headache following botulinum toxin injections administered to different anatomical regions.

**Methods:**

This prospective observational study included 102 adult patients undergoing abobotulinumtoxinA injections to the upper facial regions, masseter muscles, and/or axillary area. Headache occurrence was assessed on day 3 by telephone, at days 10–12 during follow‐up visits, and through patient‐initiated contact within the first month. Demographic variables, prior botulinum toxin exposure, injection‐related pain, toxin dose, and headache characteristics were recorded.

**Results:**

Post‐botulinum headache occurred in 13 patients (12.7%) and was generally mild‐to‐moderate, short‐lived, and self‐limited. Headache was significantly more frequent in first‐time recipients compared with previously treated patients (45.5% vs. 8.8%, *p* = 0.005). All patients who developed headache had received injections involving the upper facial regions, whereas no headache was observed after exclusive masseter or axillary treatments. No associations were found with toxin dose, procedural pain, or systemic comorbidities.

**Conclusion:**

Post‐botulinum headache is an infrequent, benign, and transient event, predominantly affecting first‐time patients undergoing upper facial injections. These findings may aid clinicians in patient counseling and expectation management.

## 1 Introduction

Botulinum neurotoxin injections exert their effects through chemodenervation of cholinergic neurons, affecting either motor or autonomic nerve terminals and resulting in a localized reduction of skeletal muscle activity or autonomic function in target organs such as eccrine sweat glands. Several commercially available preparations of botulinum neurotoxin are currently in the market, differing in their methods of manufacture, formulation, and biological profiles [1].

Owing to these properties, botulinum neurotoxin has a broad range of clinical applications, including the treatment of facial wrinkles, hyperhidrosis, bruxism, strabismus, dystonia, migraine, and blepharospasm. When administered with appropriate indications and proper injection techniques, botulinum toxin is widely regarded as an effective and safe therapeutic modality [2, 3, 4].

Despite its favorable safety profile, botulinum toxin injections are not entirely free of adverse effects. Common short‐term complications following botulinum toxin administration include injection‐site pain, edema, ecchymosis, purpura, transient hypesthesia, short‐term postinjection headaches, and less frequently, prolonged migraine attacks [5].

Postinjection headaches have been described in two main clinical forms. Minor headaches are more common and are typically self‐limited, responding well to standard over‐the‐counter analgesics. In contrast, severe postinjection headaches are rare but may be persistent and require more aggressive management, including stronger analgesics or oral corticosteroids when necessary [6].

Interestingly, botulinum neurotoxin is also an established and effective prophylactic treatment for chronic migraine [7]. However, evidence supporting its efficacy in other primary headache disorders, such as tension‐type headache and cluster headache, remains limited and inconsistent [8, 9, 10].

In this context, the occurrence of headache as a potential adverse effect following cosmetic botulinum toxin injections presents a paradoxical and clinically intriguing phenomenon, particularly given the toxin's established role in headache prophylaxis. The present prospective observational study aimed to evaluate the incidence and temporal characteristics of headache following cosmetic botulinum toxin injections administered to different anatomical regions, including the upper facial area, masseter muscles, and axillary region. In addition, we sought to examine the association between injection‐related pain and the subsequent development of headache, as well as the potential influence of a pre‐existing history of headache.

## 2 Materials and Methods

### 2.1 Study Design and Ethical Approval

This prospective observational study was conducted in patients undergoing cosmetic and medical botulinum toxin type A (BoNT‐A) injections at a single outpatient center. This study was approved by the local Institutional Review Board. The study was conducted in accordance with the principles of the Declaration of Helsinki. Written informed consent was obtained from all participants prior to inclusion in the study.

### 2.2 Study Population

Adult patients (≥ 18 years) who received botulinum toxin injections for aesthetic and functional indications were eligible for inclusion. Exclusion criteria included the presence of a known neurological disorder, chronic headache requiring continuous medical treatment, or incomplete follow‐up data. Patients receiving botulinum toxin for therapeutic neurological indications were not included.

### 2.3 Botulinum Toxin Injection Protocol

All injections were performed by two physicians, each with a minimum of 9 years of experience in cosmetic botulinum toxin applications. AbobotulinumtoxinA (Dysport) was used in all patients and administered according to routine cosmetic practice using standardized techniques.

The toxin was reconstituted with 3 mL of preservative‐free normal saline using single‐use ampoules and prepared immediately before administration. Injection volumes were standardized such that 0.1 mL corresponded to 16.6 units. No prophylactic analgesics were administered before or immediately after the procedure.

Injection sites included the upper facial regions (frontalis, glabellar complex, periorbital area, bunny lines, and perioral region), masseter muscles, and axillary area, either alone or in combination, depending on individual patient needs and aesthetic indications. The total botulinum toxin dose and treated anatomical regions were determined on an individual basis.

### 2.4 Data Collection and Baseline Assessment

Baseline demographic and clinical data were recorded for all patients, including age, sex, body mass index, smoking status, presence of systemic comorbidities, and self‐reported history of migraine. Previous exposure to botulinum toxin was documented and categorized as first‐time injection, one prior injection, or two or more prior injections.

Procedural pain intensity was assessed immediately after the procedure using a visual analog scale (VAS; 0–10).

### 2.5 Follow‐Up and Assessment of Post‐Botulinum Headache

Patients were prospectively monitored for the development of headache following botulinum toxin administration using a structured follow‐up protocol. On post‐procedural day 3, patients were contacted by telephone and specifically questioned regarding the presence of headache. A second evaluation was performed during the routine follow‐up visit on days 10–12 after the procedure. In addition, all patients were instructed to contact the clinic by telephone if headache occurred within the first month following injection.

Headache occurrence was recorded as a binary outcome (present/absent). For patients reporting headache, detailed characteristics were documented using a standardized patient follow‐up form, including headache onset day, duration, pain intensity (VAS), localization, pain type, pain course, potential triggering factors, and use of analgesic medication.

### 2.6 Adverse Events

Local adverse effects were prospectively recorded and classified as injection‐site erythema, tenderness, or numbness. Systemic adverse events, including nausea, vomiting, fever, fatigue, or malaise, were also recorded during the follow‐up period.

### 2.7 Statistical Analysis

Statistical analyses were performed using IBM SPSS Statistics (IBM Corp., Armonk, NY, USA). Continuous variables were assessed for normality using the Shapiro–Wilk test. Normally distributed variables were presented as mean ± standard deviation, whereas non‐normally distributed variables were expressed as median with interquartile range (IQR). Categorical variables were presented as number and percentage.

Comparisons between patients with and without post‐botulinum headache were performed using the independent‐samples *t*‐test for normally distributed continuous variables and the Mann–Whitney U test for non‐normally distributed continuous variables. Categorical variables were compared using the chi‐squared test or Fisher's exact test, as appropriate. A two‐sided *p* value < 0.05 was considered statistically significant.

## 3 Results

### 3.1 Study Population

A total of 102 patients were included in the final analysis. Post‐botulinum headache developed in 13 patients (12.7%), while 89 patients (87.3%) did not report headache following the procedure. The mean age of the study population was 43.4 ± 10.2 years (median: 43.5, IQR: 37.0–49.8, range: 24–70 years). The age distribution did not significantly deviate from normality according to the Shapiro–Wilk test (*p* = 0.099). Baseline demographic and clinical characteristics of patients with and without post‐botulinum headache are presented in Table 1.

**TABLE 1:** Baseline demographic and clinical characteristics according to post‐botulinum headache.

Variable	No headache (*n* = 89)	Headache (*n* = 13)	*p*
Age (years), mean ± SD	43.6 ± 10.2	42.1 ± 9.9	0.606*
BMI, median (IQR)	23.0 (21.1–26.0)	22.5 (20.6–27.3)	0.825^†^
Procedural pain (VAS), median (IQR)	4.0 (2.0–6.0)	4.0 (3.0–4.0)	0.588^†^
Total botulinum toxin dose (units), median (IQR)	132.8 (132.8–166.0)	149.4 (132.8–149.4)	0.895^†^
Previous botulinum toxin exposure, *n* (%)			0.005^‡^
No (first‐time)	6 (6.7%)	5 (38.5%)	
Yes (≥ 1 prior injection)	83 (93.3%)	8 (61.5%)	
Sex, *n* (%)			0.366^‡^
Female	55 (61.8%)	10 (76.9%)	
Male	34 (38.2%)	3 (23.1%)	
Smoking status, *n* (%)			0.555^‡^
No	44 (49.4%)	8 (61.5%)	
Yes	45 (50.6%)	5 (38.5%)	
Migraine history, *n* (%)			0.730^‡^
No	68 (76.4%)	9 (69.2%)	
Yes	21 (23.6%)	4 (30.8%)	
Systemic disease, *n* (%)			0.783^‡^
None	60 (67.4%)	11 (84.6%)	
Cardiovascular	7 (7.9%)	0 (0%)	
Endocrine	16 (18.0%)	1 (7.7%)	
Respiratory	1 (1.1%)	0 (0%)	
Allergic	3 (3.4%)	1 (7.7%)	
Psychiatric	1 (1.1%)	0 (0%)	
Renal	1 (1.1%)	0 (0%)	

*Note:* Values are presented as mean ± standard deviation (SD), median (interquartile range [IQR]) or number (%), as specified for each variable. *p* Values were calculated using the *independent‐samples *t*‐test, ^†^Mann–Whitney *U* test, and ^‡^chi‐squared test or Fisher's exact test, as appropriate.

### 3.2 Comparison of Patients With and Without Post‐Botulinum Headache

No statistically significant differences were observed between patients who developed headache and those who did not with regard to age, sex, body mass index, smoking status, and presence of systemic disease (all *p* > 0.05, Table 1). A history of migraine, recorded based on patient self‐report, was more frequently observed among patients who developed headache; however, this difference did not reach statistical significance (*p* > 0.05, Table 1).

The total administered botulinum toxin dose (expressed as total units) was comparable between the two groups (*p* > 0.05). Headache occurrence differed significantly according to prior botulinum toxin exposure. Post‐botulinum headache was observed in 5 of 11 patients (45.5%) undergoing their first botulinum toxin injection, compared with 1 of 8 patients (12.5%) with a single prior injection and 7 of 83 patients (8.4%) with two or more prior injections (*p* = 0.002, chi‐squared test). When patients were dichotomized as first‐time versus previously treated, headache was significantly less frequent among patients with prior botulinum toxin exposure (45.5% vs. 8.8%, *p* = 0.005, Fisher's exact test). Procedural pain intensity did not show a statistically significant difference between groups, with identical median VAS scores of 4.0 (*p* = 0.588, Table 1).

Botulinum toxin injections were administered to the upper facial regions, masseter muscles, and axillary area. Overall, injections involved the upper facial regions in 94 patients (92.2%), the axillary area in 18 patients (17.6%), and the masseter muscles in 5 patients (4.9%), with some patients receiving injections at more than one site. Notably, all patients who developed post‐botulinum headache (13/13, 100%) had received injections involving the upper facial regions. In contrast, no headache was observed among patients who received injections exclusively to the axillary area or the masseter muscles.

Local adverse effects were uncommon and generally mild. Injection‐site erythema was reported in 10 patients (9.8%), and localized tenderness in 1 patient (1.0%); all local reactions were transient and resolved spontaneously without medical intervention. The frequency of local adverse effects did not differ between patients with and without post‐botulinum headache. No systemic adverse events, including nausea, vomiting, fever, fatigue, or malaise, were observed in any patient during follow‐up.

### 3.3 Characteristics of Post‐Botulinum Headache

Characteristics of post‐botulinum headache among affected patients are summarized in Table 2. Headache intensity was moderate, with a median VAS score of 4.0 (IQR: 2.8–6.0). The most common headache localization was frontal (69.2%), followed by occipital, temporal, and diffuse patterns. Pain was most frequently described as throbbing or pressure‐type, and headache course was reported as either continuous or intermittent in the majority of patients. Only three patients (23.1%) required analgesic medication during the headache episode.

**TABLE 2:** Characteristics of post‐botulinum headache (*n* = 13).

Characteristic	Value
Pain intensity (VAS), median (IQR)	4.0 (2.8–6.0)
Pain localization, *n* (%)	
Frontal	9 (69.2%)
Occipital	2 (15.4%)
Temporal	1 (7.7%)
Diffuse	1 (7.7%)
Pain type, *n* (%)	
Throbbing	3 (23.1%)
Pressure type	3 (23.1%)
Diffuse	2 (15.4%)
Aching	2 (15.4%)
Tension type	2 (15.4%)
Explosive	1 (7.7%)
Pain course, *n* (%)	
Continuous	5 (38.5%)
Intermittent	5 (38.5%)
Fluctuating	2 (15.4%)
Continuous with attacks	1 (7.7%)
Analgesic use, *n* (%)	3 (23.1%)

*Note:* Values are presented as median (interquartile range) or number (%), as appropriate. Analyses were descriptive and limited to patients who developed post‐botulinum headache.

Among patients who developed post‐botulinum headache (*n* = 13), headache onset occurred on day 1 in six patients (46.2%), on day 2 in six patients (46.2%), and on day 3 in one patient (7.7%) following injection. The median time to headache onset was 2 days (IQR, 1–2 days; range, 1–3 days). Headache duration was short‐lived in most cases, lasting 1 day in 12 patients (92.3%) and 2 days in 1 patient (7.7%), with a median duration of 1 day (IQR, 1–1 days; range, 1–2 days). Specific triggering factors were uncommon. Headache exacerbation was reported with loud noise in one patient and with upward gaze in another patient.

## 4 Discussion

In this prospective observational study, post‐botulinum headache was observed in 12.7% of patients undergoing cosmetic botulinum toxin injections. Headaches were generally mild‐to‐moderate in intensity, short‐lived, and self‐limited, with most resolving within 1–2 days. The most prominent finding of our study was the significantly higher headache incidence among first‐time botulinum toxin recipients, whereas no associations were identified with demographic characteristics, systemic comorbidities, total toxin dose, or injection‐related pain intensity.

Botulinum toxin is primarily used for the treatment of dynamic wrinkles and is widely regarded as a safe and effective cosmetic intervention. Among the available serotypes (A–G), BoNT‐A is the most commonly used, with several commercially available formulations, including onabotulinumtoxinA, abobotulinumtoxinA, and incobotulinumtoxinA. The most frequently reported adverse effects are mild and transient injection‐site reactions such as bruising, ecchymosis, and localized pain [11, 12].

Headache is a recognized short‐term adverse effect following cosmetic botulinum toxin injections. Proposed mechanisms include transient muscle spasm immediately after injection followed by toxin‐induced relaxation, as well as local factors such as periosteal irritation or deep muscle hematoma formation [13]. Notably, botulinum toxin is also an established and effective prophylactic treatment for chronic migraine, which creates a paradoxical situation when headache occurs after cosmetic applications [4]. In migraine prophylaxis, its analgesic effect is thought to result from inhibition of neurotransmitter release, including glutamate, substance P, and calcitonin gene‐related peptide, through interaction with the SNARE complex [7].

In our cohort, the observed headaches were predominantly frontal, temporal, occipital, or diffuse in location and described as throbbing or pressure‐type. Importantly, pain was not confined to injection sites and resolved rapidly, findings that argue against periosteal irritation or muscle hematoma as primary mechanisms [13]. The benign and self‐limited course observed in our study is consistent with prior reports indicating that post‐botulinum headaches typically resolve within 24–72 h [14]. Systemic symptoms were rare, in line with previous safety reviews [15].

Our findings are largely consistent with previous observational studies. In a retrospective study by Muallaaziz et al., headache onset occurred predominantly within the first 24 h after injection, and the majority of cases resolved within 3 days, with most patients requiring no analgesic treatment [16]. Similar to their results, we found no association between headache occurrence and administered toxin dose. However, important differences should be noted. Our study was conducted prospectively and exclusively utilized abobotulinumtoxinA, whereas Muallaaziz et al. evaluated patients treated with onabotulinumtoxinA. These formulations differ in molecular structure, diffusion characteristics, and biological behavior, which may influence adverse event profiles.

A distinctive strength of our study is the inclusion of patients receiving botulinum toxin injections to non‐facial regions, including the masseter muscles and axillary area. Notably, no headaches were observed among patients treated exclusively in these regions, despite higher toxin doses being administered for axillary hyperhidrosis compared with upper facial applications. This finding suggests that post‐procedural headache may be more closely related to anatomical location and trigeminal system involvement rather than systemic toxin exposure.

We also observed no significant association between post‐procedural headache and systemic comorbidities. This contrasts with findings from Muallaaziz et al., who reported a significant association with hypertension. Differences in study design, toxin formulation, and patient populations may partially explain this discrepancy. Prospective studies directly comparing different botulinum toxin formulations are warranted to further clarify the potential role of systemic conditions in headache development following cosmetic injections.

The markedly higher headache rate observed among first‐time recipients suggests that exposure‐related factors may be involved. A novel nociceptive stimulus associated with facial injections, particularly within trigeminally innervated regions, may transiently increase central nociceptive excitability, thereby lowering the threshold for headache generation in susceptible individuals [17]. Psychological factors such as anticipatory anxiety and heightened vigilance during an unfamiliar cosmetic procedure may further contribute through stress‐related pathways and increased pericranial muscle tension, even when procedural pain intensity is comparable [18]. Conversely, the lower headache frequency observed among previously treated patients may reflect habituation with repeated exposure, involving improved tolerability, reduced local tissue reactivity, attenuation of neurogenic inflammatory responses, and diminished stress‐related responses due to procedural familiarity [19, 20].

This study has several limitations that should be acknowledged. Although its prospective design strengthens the reliability of data collection, the number of patients who developed post‐procedural headache was relatively small, which may have limited the statistical power to detect associations with less common risk factors. Headache characteristics were assessed based on patient self‐report, introducing the potential for reporting bias. In addition, psychological variables such as anxiety were not evaluated using standardized instruments, precluding a direct assessment of their contribution to headache development. Finally, this was a single‐center study using a single botulinum toxin formulation (abobotulinumtoxinA), which may limit the generalizability of the findings.

## 5 Conclusion

Post‐botulinum headache occurred in 12.7% of patients after cosmetic abobotulinumtoxin A injections and was generally mild‐to‐moderate, short‐lived, and self‐limited. Headache was significantly more frequent in first‐time recipients and was observed only in patients receiving injections involving the upper facial regions, whereas no headaches occurred after exclusive masseter or axillary treatments. These findings may assist clinicians in counseling patients—particularly those undergoing their first upper facial botulinum toxin procedure—regarding the likelihood and expected course of post‐procedural headache.

## Author Contributions

Ümit Akpınar conceived and designed the study, performed the botulinum toxin procedures, supervised data acquisition, conducted the statistical analysis and interpretation of the data, and drafted and finalized the manuscript. Ömer Vural contributed to the study design, performed the statistical analysis, and contributed to the drafting of the manuscript. Alper Köycü contributed to the study design and drafting of the manuscript. Ekrem Civaş contributed to the botulinum toxin procedures and study design.

## Funding

The authors have nothing to report.

## Disclosure

Additional Information: The data that support the findings of this study are available from the corresponding author upon reasonable request. Language refinement and grammatical editing were partially assisted using OpenAI's ChatGPT. All AI‐generated suggestions were critically reviewed, edited, and approved by the authors, who take full responsibility for the final content.

## Ethics Statement

This study was approved by the Ethics Committee of Başkent University Institutional Review Board (approval number: KA25/472). The study was conducted in accordance with the principles of the Declaration of Helsinki.

## Consent

Written informed consent was obtained from all participants prior to inclusion in the study.

## Conflicts of Interest

The authors declare no conflicts of interest.

## Data Availability

The data that support the findings of this study are available from the corresponding author upon reasonable request.
